# Splenic Enlargement During Neoadjuvant Chemotherapy for Gastric Cancer: Treatment-Related Phenomenon or Prognostic Biomarker?

**DOI:** 10.3390/jcm15218303

**Published:** 2026-10-25

**Authors:** Ezgi Altınsoy, Veysel Cem Özcan, Abdurrahman Başpınar, Başak Gülpınar, Selim Tamam, Serdar Çulcu, Salim Demirci

**Affiliations:** 1 Surgical Oncology Department, Van Training and Research Hospital, Süphan Mahallesi Hava Yolu Kavşağı 1. Kilometre, Edremit 65300, Van, Türkiye; ezgialtinsoy@gmail.com; 2 Surgical Oncology Department, Antalya City Hospital, Göçerler Mahallesi 5379. Sokak, Kepez 07080, Antalya, Türkiye; cemzcan@gmail.com; 3 Surgical Oncology Department, Ordu University Training and Research Hospital, Bucak Mahallesi Nefsi Bucak Caddesi, Altınordu 52200, Ordu, Türkiye; abdurrahmanbaspinar1@gmail.com; 4 Radiology Department, Faculty of Medicine, Ankara University, Balkiraz Mahallesi Tıp Fakültesi Caddesi, Mamak 06620, Ankara, Türkiye; basak.gulpinar@yahoo.com; 5 Surgical Oncology Department, Faculty of Medicine, Ankara University, Balkiraz Mahallesi Tıp Fakültesi Caddesi, Mamak 06620, Ankara, Türkiye; selimtamam@hotmail.com (S.T.); salimdemirci@yahoo.com (S.D.)

**Keywords:** gastric cancer, neoadjuvant chemotherapy, spleen volume, splenomegaly, treatment response

## Abstract

**Introduction:** Spleen volume (SV) may increase during systemic chemotherapy, potentially reflecting treatment-related hepatic, hematological, and immune alterations. However, the clinical significance of SV changes during neoadjuvant chemotherapy (NAC) for gastric cancer remains unclear. This study evaluated SV changes during NAC and their associations with radiological response and overall survival (OS). **Methods:** This retrospective cohort study included 111 patients with gastric adenocarcinoma who received NAC followed by curative-intent gastrectomy. SV was measured on computed tomography before and after NAC using semiautomated volumetric segmentation. Relative SV change (ΔSV%) was calculated. Radiological response was assessed according to RECIST 1.1. Associations of ΔSV% with objective response and OS were evaluated using logistic and Cox regression analyses, respectively. **Results:** Mean SV increased significantly from 250.59 ± 98.11 to 335.40 ± 126.81 cm^3^ (*p* < 0.001), with a mean ΔSV% of 34.53 ± 15.72%. SV increased in 108 patients (97.3%). Objective response was observed in 56 patients (50.5%). ΔSV% did not differ between responders and nonresponders (34.05 ± 15.66% vs. 35.03 ± 15.90%; *p* = 0.730) and was not associated with objective response after multivariable adjustment (OR per 10% increase, 0.932; 95% CI, 0.715–1.207; *p* = 0.578). ΔSV% was also not associated with OS (adjusted HR, 1.031; 95% CI, 0.859–1.236; *p* = 0.745). **Conclusions:** SV increased markedly in nearly all patients during NAC for gastric cancer. However, the magnitude of this increase was not associated with radiological response or OS, suggesting that splenic enlargement during NAC may represent a treatment-associated phenomenon rather than a marker of antitumor efficacy or prognosis.

## 1. Introduction

Gastric cancer remains a major cause of cancer-related morbidity and mortality worldwide and is frequently diagnosed at an advanced stage [1]. Although surgical resection remains the cornerstone of curative treatment, surgery alone is associated with a considerable risk of recurrence. Consequently, multimodal treatment strategies incorporating systemic chemotherapy have become an integral component of the management of resectable locally advanced gastric cancer, and perioperative chemotherapy is currently widely adopted in clinical practice [2].

Neoadjuvant chemotherapy (NAC) may facilitate tumor downstaging, enable early treatment of micrometastatic disease, and provide an opportunity to assess tumor response to systemic therapy before surgical resection [2]. Nevertheless, its effects are not confined to the tumor and may involve treatment-related alterations in several organs and systemic biological processes. Oxaliplatin, which constitutes a key component of contemporary perioperative regimens for gastric cancer, has been associated with hepatic sinusoidal injury and sinusoidal obstruction syndrome [3]. The resulting increase in portal venous pressure may lead to splenic enlargement and thrombocytopenia, and treatment-related changes in spleen volume (SV) have consequently been investigated as a non-invasive surrogate marker of oxaliplatin-associated hepatic sinusoidal injury [3,4].

The potential significance of SV, however, may extend beyond chemotherapy-related toxicity. The spleen has an important role in hematopoietic, inflammatory, and immune regulation, providing a biological rationale for investigating volumetric alterations as a marker of systemic host response to cancer and anticancer treatment [5]. Both baseline SV and treatment-related changes in SV have therefore been evaluated in different malignancies in relation to treatment response and oncological outcomes [6,7]. However, data regarding SV dynamics during NAC for gastric cancer and their clinical relevance remain limited [7]. Whether treatment-related splenic enlargement predominantly reflects a systemic effect of treatment or is associated with tumor response and subsequent prognosis has not been clearly established.

The present study therefore aimed to quantify changes in SV during NAC in patients with gastric cancer and to evaluate the association of these changes with radiological treatment response and overall survival (OS). In addition, associations between treatment-related SV changes and selected hematological and systemic inflammatory parameters were explored.

## 2. Methods

### 2.1. Study Design and Patient Population

This retrospective cohort study included patients with gastric adenocarcinoma who were treated at the Department of Surgical Oncology, Ankara University Faculty of Medicine, between 2016 and 2025. Clinical, laboratory, radiological, and follow-up data were retrospectively retrieved from institutional medical records. The study was approved by the Ankara University Faculty of Medicine Clinical Research Ethics Committee (Application No. 2026000469; Registration No. 2026/469). All patients had provided institutional consent for the use of their clinical data for scientific research purposes at the initiation of treatment.

Patients aged ≥18 years with histologically confirmed gastric adenocarcinoma who received NAC and subsequently underwent curative-intent gastrectomy at our institution were eligible for inclusion. The indication for NAC was determined by the institutional multidisciplinary tumor board in accordance with contemporary clinical guidelines [2].

Patients were excluded if they had a histological diagnosis other than adenocarcinoma, had metastatic disease at initial diagnosis or following NAC, underwent palliative gastrectomy, lacked evaluable pre- or post-NAC computed tomography (CT) imaging, were considered medically unfit for surgery after NAC, or had insufficient follow-up data.

### 2.2. Neoadjuvant Treatment and Surgical Management

NAC regimens were selected according to contemporary clinical guidelines and multidisciplinary tumor board recommendations during the study period. Treatment predominantly consisted of fluorouracil, leucovorin, oxaliplatin, and docetaxel (FLOT) or fluorouracil, leucovorin, and oxaliplatin (FOLFOX), while alternative fluoropyrimidine-based combination regimens were administered in a small number of patients.

Following NAC, patients underwent total or subtotal gastrectomy according to tumor location and extent. D2 lymph node dissection was performed in all patients.

### 2.3. Data Collection and Outcomes

Demographic and clinical variables, including age, sex, body mass index (BMI), American Society of Anesthesiologists (ASA) physical status, Eastern Cooperative Oncology Group (ECOG) performance status, clinical T and N stage, chemotherapy regimen, and number of NAC cycles, were retrospectively retrieved from institutional records. Post-NAC preoperative laboratory parameters, including platelet count, neutrophil and lymphocyte counts, C-reactive protein (CRP), and albumin levels, were recorded. The neutrophil-to-lymphocyte ratio (NLR) was calculated by dividing the absolute neutrophil count by the absolute lymphocyte count, and the CRP-to-albumin ratio (CAR) was calculated by dividing serum CRP (mg/L) by serum albumin (g/dL).

Clinical and pathological staging was performed according to the eighth edition of the American Joint Committee on Cancer/Union for International Cancer Control (AJCC/UICC) TNM staging system. For analysis, the number of NAC cycles was categorized as 1–3, 4, or ≥5 cycles.

Postoperative pathological data included histological subtype according to the Lauren classification and pathological T (ypT) and N (ypN) categories. Histological grade and tumor regression grade were not systematically documented throughout the study period and were therefore not included as study variables.

Radiological response to NAC was evaluated by an abdominal radiologist with 12 years of experience according to the Response Evaluation Criteria in Solid Tumors (RECIST), version 1.1 [8]. The primary gastric tumor and pathological lymph nodes were evaluated together as target lesions. All patients included in the cohort had measurable target disease. For RECIST assessment, paired pre- and post-NAC CT examinations from each patient were reviewed together. Patients were classified as having complete response (CR), partial response (PR), stable disease (SD), or progressive disease (PD). CR and PR were categorized as objective responders, whereas SD and PD were categorized as nonresponders.

OS was defined as the interval from the date of diagnosis to death from any cause or last available follow-up for patients who remained alive. The date of diagnosis was selected as a uniform clinical starting point preceding NAC for all patients. The final follow-up date was 31 December 2025. Postoperative follow-up was routinely performed every 3 months during the first 2 years after surgery and every 6 months thereafter up to 5 years. Follow-up visits included clinical examination and laboratory assessment. Thoracoabdominal CT was routinely performed at 6-month intervals during follow-up.

### 2.4. CT Acquisition and Spleen Volume Measurement

Pre-NAC CT examinations were performed at diagnosis, whereas post-NAC CT examinations were obtained 4–6 weeks after completion of NAC during the preoperative period. CT images were acquired using a 64-detector row multidetector CT scanner (Aquilion 64; Toshiba Medical Systems, Tokyo, Japan). All examinations were performed on the same scanner using a standardized single-phase contrast-enhanced abdominal CT protocol.

Images were acquired with a slice thickness of 5 mm, reconstruction interval of 1 mm, tube voltage of 120 kV, automatic tube current modulation, and tube rotation time of 0.5 s. The scanning range extended from the diaphragmatic dome to the lesser trochanters in the craniocaudal direction. A nonionic iodinated contrast agent (350 mg iodine/mL) was administered intravenously at a dose of 2 mL/kg through a 22-gauge catheter placed in an antecubital vein at a rate of 3.5 mL/s using an automatic power injector. Portal venous phase images were acquired approximately 70 s after contrast administration.

Spleen volumetric measurements were performed by the same abdominal radiologist who evaluated radiological treatment response. Splenic volumetric measurements were performed by the same radiologist in a separate reading session from the RECIST assessment. For spleen volumetry, paired pre- and post-NAC CT examinations from each patient were reviewed together. The volumetric assessment was performed independently of clinical, laboratory, and survival outcome data. SV was measured using Vitrea 4.0 post-processing software (Vital Images, Minnetonka, MN, USA). Voxel-based volumetric analysis was performed using a semiautomated threshold-based segmentation algorithm that differentiated splenic tissue from surrounding structures. Following selection of an initial region of interest, voxels with attenuation values similar to those of the splenic parenchyma were automatically identified. When adjacent tissues with similar attenuation values were inadvertently included, their contours were manually delineated on axial images and excluded from the segmented volume. Total splenic parenchymal volume was subsequently calculated automatically and expressed in cubic centimeters (cm^3^).

SV was measured on CT examinations obtained before initiation and after completion of NAC. The relative change in SV (ΔSV%) was calculated as follows:

ΔSV% = [(post-NAC SV − pre-NAC SV)/pre-NAC SV] × 100.

### 2.5. Statistical Analysis

Statistical analyses were performed using IBM SPSS Statistics for Windows, version 31.0 (IBM Corp., Armonk, NY, USA). Normality of continuous variables was assessed using skewness and kurtosis coefficients, with values within ±1.96 considered compatible with a normal distribution. Continuous variables are presented as mean ± standard deviation or median (minimum–maximum), as appropriate, and categorical variables as number and percentage. Pre- and post-NAC spleen volumes were compared using the paired-samples *t* test.

For comparisons between objective responders (CR/PR) and nonresponders (SD/PD), the independent-samples *t* test or Mann–Whitney U test was used for continuous variables and Pearson’s chi-square or Fisher’s exact test for categorical variables, as appropriate. Factors associated with objective response were evaluated using binary logistic regression analysis. Variables considered clinically relevant to the respective outcome and selected variables identified in univariable analyses were included in the multivariable models. ΔSV% was evaluated as a continuous variable and expressed per 10-percentage-point increase in regression analyses. Results are reported as odds ratios (ORs) with 95% confidence intervals (CIs).

Survival curves were estimated using the Kaplan–Meier method and compared using the log-rank test. For Kaplan–Meier analysis only, ΔSV% was dichotomized at its median value (≤38.14% vs. >38.14%). Factors associated with OS were evaluated using Cox proportional hazards regression analysis, with results reported as hazard ratios (HRs) and 95% CIs. A two-sided *p* value < 0.05 was considered statistically significant. Median follow-up duration was estimated using the reverse Kaplan–Meier method.

Exploratory associations between ΔSV% and post-NAC preoperative laboratory parameters were assessed using Spearman rank correlation analysis.

In exploratory analyses, pre-NAC SV and ΔSV% were compared across postoperative ypT and ypN categories using the Kruskal–Wallis test.

## 3. Results

### 3.1. Patient Characteristics and Radiological Response

A total of 111 patients with gastric adenocarcinoma who received NAC and subsequently underwent curative-intent gastrectomy were included in the study. The mean age was 60.37 ± 9.73 years, and 77 patients (69.4%) were male. Most patients had locally advanced disease at presentation; 98 (88.3%) had cT3–4 tumors, and 90 (81.1%) had clinically positive lymph nodes. FLOT was the predominant NAC regimen, administered to 97 patients (87.4%), while 10 (9.0%) received FOLFOX and 4 (3.6%) received other regimens. Most patients (*n* = 87, 78.4%) received four cycles of NAC. Demographic, clinical, treatment, laboratory, and SV characteristics are summarized in Table 1.

According to RECIST 1.1, CR was observed in 3 patients (2.7%), PR in 53 (47.7%), SD in 41 (36.9%), and PD in 14 (12.6%). Accordingly, 56 patients (50.5%) were classified as objective responders (CR/PR) and 55 (49.5%) as nonresponders (SD/PD).

According to the Lauren classification, 79 patients (71.2%) had intestinal-type, 28 (25.2%) diffuse-type, and 3 (2.7%) mixed-type tumors; one patient (0.9%) had no residual tumor in the surgical specimen and could not be classified according to Lauren subtype. Postoperative ypT distribution differed significantly between responders and nonresponders (*p* = 0.005), with lower ypT categories more frequently observed among responders, whereas the ypN distribution did not differ significantly between the groups (*p* = 0.088) (Table 1).

In exploratory analyses, neither pre-NAC SV nor ΔSV% differed significantly across postoperative ypT categories (*p* = 0.092 and *p* = 0.893, respectively) or ypN categories (*p* = 0.973 and *p* = 0.515, respectively).

### 3.2. Changes in Spleen Volume During Neoadjuvant Chemotherapy

Mean SV increased from 250.59 ± 98.11 cm^3^ before NAC to 335.40 ± 126.81 cm^3^ after NAC (paired-samples *t* test, *p* < 0.001). The mean ΔSV% was 34.53 ± 15.72%, and the median ΔSV% was 38.14% (range, −22.83% to 83.20%) (Table 1).

An increase in SV was observed in 108 of 111 patients (97.3%). Increases exceeding 20%, 30%, and 50% of pre-NAC SV were observed in 94 (84.7%), 84 (75.7%), and 10 (9.0%) patients, respectively.

In exploratory correlation analyses, ΔSV% was not significantly associated with post-NAC platelet count (ρ = 0.012, *p* = 0.904), NLR (ρ = 0.046, *p* = 0.633), or CAR (ρ = 0.049, *p* = 0.609).

### 3.3. Spleen Volume Change and Objective Tumor Response

Pre-NAC SV did not differ significantly between responders and nonresponders (248.12 ± 102.03 vs. 253.11 ± 94.82 cm^3^; *p* = 0.682). Similarly, post-NAC SV was comparable between the two groups (328.88 ± 125.34 vs. 342.04 ± 129.10 cm^3^; *p* = 0.332). The magnitude of SV change was also similar, with a mean ΔSV% of 34.05 ± 15.66% among responders and 35.03 ± 15.90% among nonresponders (*p* = 0.730) (Table 1). Post-NAC preoperative platelet count, NLR, and CAR also did not differ significantly between responders and nonresponders.

Responders were older than nonresponders (62.87 ± 9.49 vs. 57.82 ± 9.39 years; *p* = 0.006), whereas no significant differences were observed in sex, BMI, ECOG performance status, clinical stage, nodal status, NAC regimen, or number of chemotherapy cycles (Table 1).

In univariable logistic regression analysis, ΔSV% was not associated with objective response (OR per 10-percentage-point increase, 0.961; 95% CI, 0.757–1.220; *p* = 0.743). This association remained nonsignificant after adjustment for age, sex, BMI, ECOG performance status, and clinical stage (adjusted OR 0.932, 95% CI 0.715–1.207; *p* = 0.578). Increasing age was independently associated with higher odds of objective response (adjusted OR 1.077 per year, 95% CI 1.027–1.130; *p* = 0.002), whereas patients with ECOG 1–2 had lower odds of response compared with those with ECOG 0 (adjusted OR 0.304, 95% CI 0.120–0.771; *p* = 0.012). Univariable and multivariable logistic regression analyses are presented in Table 2.

### 3.4. Spleen Volume Change and Overall Survival

**Figure 1:**
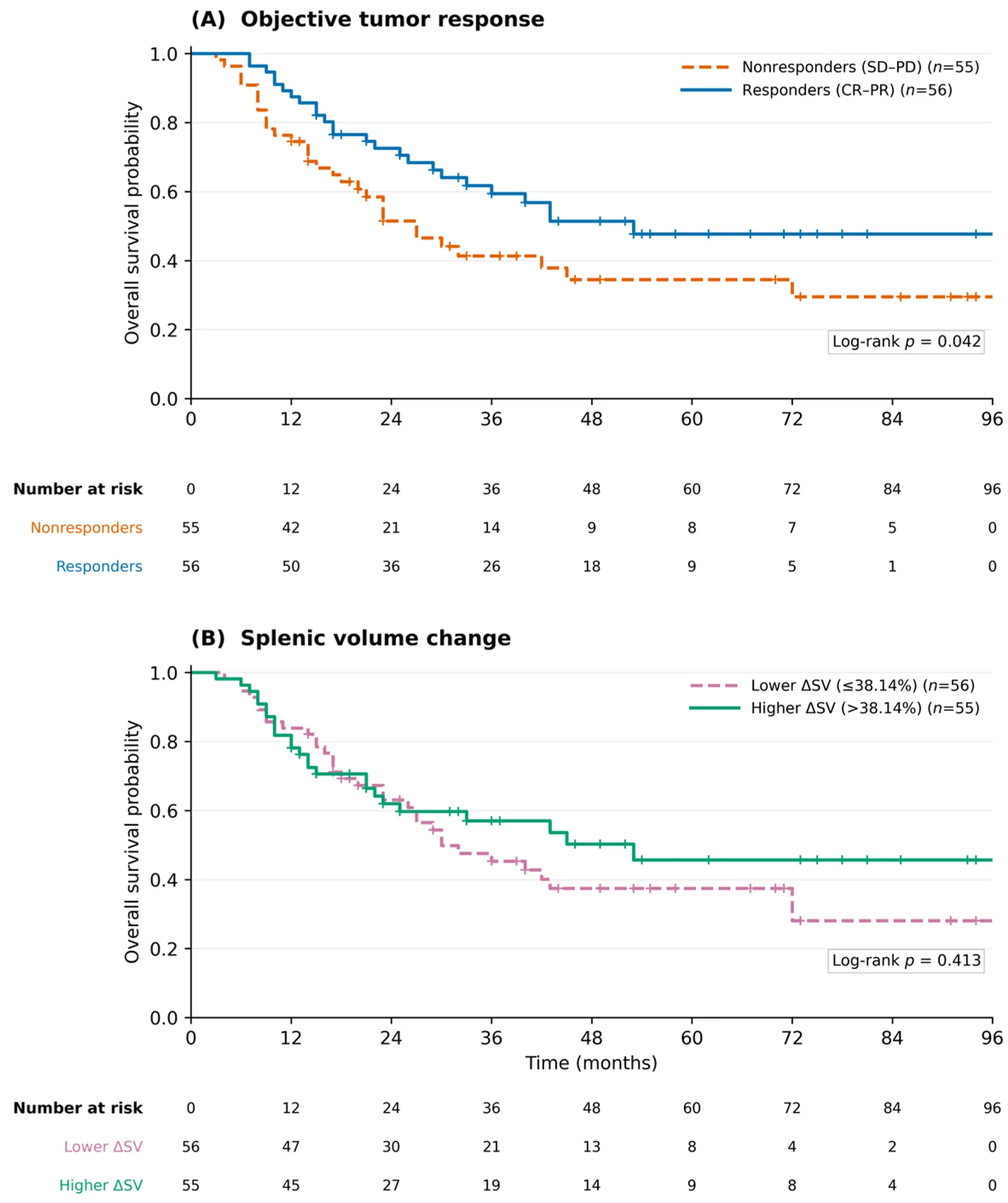
Kaplan–Meier curves for overall survival according to objective tumor response and change in spleen volume: (**A**) Overall survival according to radiological response to neoadjuvant chemotherapy: responders (CR/PR; *n* = 56) versus nonresponders (SD/PD; *n* = 55) (log-rank *p* = 0.042). (**B**) Overall survival according to spleen volume change dichotomized at the cohort median of 38.14%: lower change (≤38.14%; *n* = 56) versus higher change (>38.14%; *n* = 55) (log-rank *p* = 0.413). CR, complete response; PR, partial response; SD, stable disease; PD, progressive disease.

The median follow-up duration, estimated using the reverse Kaplan–Meier method, was 49 months, with a final follow-up date of 31 December 2025. During follow-up, 57 patients (51.4%) died. Kaplan–Meier analysis demonstrated significantly longer OS among patients with an objective response to NAC than among nonresponders (estimated mean OS, 57.86 vs. 43.49 months; log-rank *p* = 0.042) (Figure 1A). 

In contrast, OS did not differ significantly according to the magnitude of SV change. Patients with ΔSV% above the cohort median (>38.14%) had an estimated mean OS of 54.65 months compared with 46.64 months among those with ΔSV% ≤ 38.14% (log-rank *p* = 0.413) (Figure 1B). 

Consistent with the Kaplan–Meier findings, ΔSV% was not associated with OS when evaluated as a continuous variable. Each 10-percentage-point increase in ΔSV% yielded an HR of 1.041 (95% CI 0.886–1.243; *p* = 0.603) in univariable analysis and an adjusted HR of 1.031 (95% CI 0.859–1.236; *p* = 0.745) after adjustment for age, sex, BMI, clinical stage, and objective tumor response. Pre-NAC SV was also not associated with OS (HR 1.001, 95% CI 0.998–1.003; *p* = 0.586) (Table 3).

Objective response was associated with improved OS in univariable Cox analysis (HR 0.585, 95% CI 0.346–0.990; *p* = 0.046), although the association was no longer statistically significant after multivariable adjustment (adjusted HR 0.656, 95% CI 0.372–1.155; *p* = 0.144). BMI reached statistical significance in the multivariable analysis (adjusted HR 0.925, 95% CI 0.857–0.998; *p* = 0.044); however, the overall multivariable model was not statistically significant (likelihood-ratio χ^2^ = 9.848; *p* = 0.131) (Table 3).

## 4. Discussion

The present study demonstrated a marked and highly prevalent increase in SV during NAC in patients with gastric cancer. The mean ΔSV% was 34.53%, with an increase in SV observed in 97.3% of patients. Despite the magnitude and consistency of this volumetric change, ΔSV% was not associated with radiological tumor response or OS. Pre-NAC SV was likewise not associated with OS. These findings suggest that splenic enlargement during NAC is a common treatment-related phenomenon in gastric cancer, but its magnitude does not appear to reflect radiological tumor response or provide prognostic information regarding OS in this setting.

One plausible explanation for the observed splenic enlargement is oxaliplatin-associated hepatic sinusoidal injury. Oxaliplatin-containing chemotherapy has been associated with sinusoidal obstruction syndrome, which may increase portal venous pressure and subsequently result in splenic congestion and enlargement [3]. Previous studies have therefore proposed treatment-related increases in SV as a non-invasive surrogate marker of oxaliplatin-associated hepatic sinusoidal injury [3]. Overman et al. reported splenic enlargement in 86% of patients receiving adjuvant FOLFOX, with an increase of at least 50% in 24%, and demonstrated an association between increasing spleen size and both cumulative oxaliplatin exposure and histological severity of sinusoidal injury [3]. Other studies using CT volumetry and non-invasive measures of hepatic injury have similarly supported an association between oxaliplatin exposure, sinusoidal injury, and increasing spleen volume [9,10]. This finding has also been reproduced in multicenter data, while persistent splenic enlargement after completion of oxaliplatin-based chemotherapy has been reported [11,12].

Because hepatic sinusoidal injury, portal hemodynamics, and immune-cell alterations were not directly assessed in this study, these mechanisms should be regarded as biologically plausible explanations rather than demonstrated causes of splenic enlargement in our cohort.

The magnitude of splenic enlargement observed in the present cohort is consistent with previous reports in patients receiving oxaliplatin-based chemotherapy. El Chediak et al. prospectively evaluated SV during oxaliplatin treatment and reported a median increase of 31% at three months, with splenic enlargement occurring in 74% of patients [4]. In our cohort, the mean ΔSV% was 34.53%, while 84.7% and 75.7% of patients experienced increases exceeding 20% and 30%, respectively. The particularly high prevalence of splenic enlargement in our population may partly reflect the predominance of oxaliplatin-containing treatment, as 87.4% of patients received FLOT, and an additional 9.0% received FOLFOX. Nevertheless, the retrospective design of the present study and the absence of direct assessment of hepatic sinusoidal injury preclude attributing the observed volumetric changes specifically to oxaliplatin-induced hepatotoxicity.

The relationship between splenic enlargement and hematological toxicity has not been consistent across previous studies. Increased SV has been associated with subsequent thrombocytopenia in some oxaliplatin-treated cohorts, supporting a portal hypertension–hypersplenism mechanism [13]. However, other studies have failed to demonstrate a direct relationship between spleen enlargement and thrombocytopenia or other conventional toxicity parameters [4]. In the present study, exploratory analyses did not demonstrate a meaningful correlation between ΔSV% and post-NAC platelet count. This finding should be interpreted cautiously because only the post-NAC platelet measurement was evaluated, rather than the longitudinal change from baseline. Therefore, the absence of an association with the post-NAC platelet count does not exclude an underlying hepatic sinusoidal mechanism.

The biological relevance of the spleen in cancer may, however, extend beyond chemotherapy-related hepatotoxicity. As the largest secondary lymphoid organ, the spleen participates in both innate and adaptive immune regulation and may serve as a reservoir for several immune and myeloid cell populations involved in tumor-associated inflammation. Accordingly, splenic volume has increasingly been investigated as an imaging biomarker of systemic host–tumor interactions [5]. In gastric cancer, higher baseline SV has been associated with poorer survival, lower circulating lymphocyte counts, higher neutrophil-to-lymphocyte ratios, and reduced circulating CD4+ T-cell and natural killer-cell populations [7]. The spleen may also participate in tumor–host interactions through the expansion of immunosuppressive myeloid populations, particularly myeloid-derived suppressor cells, which can accumulate in the spleen, circulation, and tumor microenvironment and contribute to suppression of antitumor immunity [6]. Similar associations between splenic enlargement, systemic immune alterations, and unfavorable outcomes have been described in other malignancies [6]. In metastatic colorectal cancer, baseline splenic volume has been associated with circulating MDSC accumulation and treatment outcomes, providing clinical support for a relationship between splenic morphology and systemic immunosuppressive myeloid activity [14]. These observations provide a biological rationale for considering splenic enlargement not merely as a consequence of portal hemodynamic changes but also as a potential marker of systemic immune and inflammatory activity.

Importantly, baseline SV and treatment-related changes in SV may represent biologically distinct phenomena. Baseline SV may reflect pre-existing host characteristics, tumor-associated inflammation, and immune status, whereas treatment-related ΔSV% may represent a composite response to therapy, including hepatic sinusoidal injury, portal hemodynamic changes, hematopoietic adaptation, and systemic inflammatory or immune effects [3,6,7]. This distinction may partly explain why baseline and dynamic spleen measurements have shown heterogeneous prognostic associations across different malignancies and treatment settings [6]. In the present study, neither pre-NAC SV nor treatment-related ΔSV% was associated with OS, suggesting limited prognostic relevance of either measure within this relatively homogeneous neoadjuvant gastric cancer cohort. 

The absence of an association between ΔSV% and objective tumor response is another relevant finding. Mean ΔSV% was almost identical between responders and nonresponders, and the association remained nonsignificant when ΔSV% was analyzed continuously in multivariable logistic regression. These findings suggest that the magnitude of splenic enlargement does not closely parallel radiological tumor regression during cytotoxic chemotherapy. While tumor shrinkage reflects the radiological response of malignant tissue to treatment, splenic remodeling may instead reflect treatment exposure and host-related physiological responses. Thus, in this setting, the magnitude of splenic enlargement should not be interpreted as an imaging surrogate of antitumor efficacy.

Our findings regarding survival are consistent with previous gastric cancer data. Zeng et al., in a large retrospective cohort, reported that high baseline SV was associated with unfavorable prognosis and altered circulating immune-cell profiles; however, among patients receiving NAC, the increase in SV during treatment was not associated with prognosis [7]. The present study extends these observations by focusing specifically on a neoadjuvant-treated gastric cancer population and by evaluating ΔSV% in relation to both RECIST-defined objective response and OS. Consistent with Zeng et al., ΔSV% was not associated with OS when modeled continuously, and dichotomization at the cohort median likewise did not identify a survival difference [7]. Taken together, these findings do not support treatment-related splenic enlargement as a surrogate marker of tumor response or as a prognostic biomarker in the neoadjuvant setting.

The prognostic significance of treatment-related SV change may also depend on treatment context. Studies of patients receiving immune checkpoint inhibitors have suggested that dynamic changes in SV may carry prognostic information, potentially reflecting alterations in systemic immune-cell populations and myeloid-derived suppressor cell-mediated immunosuppression [6]. Supporting a potential link between splenic morphology and systemic immunity, baseline SV has also been associated with circulating myeloid-derived suppressor cell accumulation and treatment outcomes in metastatic colorectal cancer [14]. More recently, dynamic SV changes have been associated with survival outcomes in patients with advanced gastric cancer receiving chemotherapy combined with immunotherapy [15]. During oxaliplatin-containing cytotoxic chemotherapy, however, splenic enlargement may be substantially influenced by hepatic sinusoidal injury and associated portal hemodynamic changes [3]. Therefore, apparently conflicting prognostic findings across studies may not necessarily be contradictory but may instead reflect treatment-specific biological mechanisms. In patients receiving NAC for gastric cancer, our findings suggest that splenic enlargement may predominantly represent a treatment-associated phenomenon rather than a marker of tumor response or prognosis, although a prognostic role in other populations or treatment settings cannot be excluded.

Objective radiological response was associated with longer OS on Kaplan–Meier analysis and in univariable Cox regression. However, this association was no longer statistically significant after multivariable adjustment. Accordingly, these findings should not be interpreted as demonstrating an independent prognostic effect of RECIST-defined objective response in this cohort. From a clinical perspective, the frequent occurrence of splenic enlargement during NAC should not, in isolation, be interpreted as evidence of favorable tumor response or adverse oncological prognosis, but rather considered within the broader context of treatment exposure and treatment-related systemic effects.

This study has several limitations. Its retrospective, single-center design limits the generalizability of the findings and precludes causal inference regarding the mechanism of splenic enlargement. Although the vast majority of patients received oxaliplatin-containing FLOT or FOLFOX, the study was not designed to distinguish oxaliplatin-specific effects from the effects of NAC more broadly. Histological confirmation of hepatic sinusoidal injury was unavailable, and direct measures of portal hypertension were not assessed. In addition, hematological and inflammatory parameters were evaluated at the preoperative time point, preventing assessment of longitudinal changes in platelet count, NLR, and CAR from baseline. Treatment exposure was relatively homogeneous, limiting the ability to evaluate regimen- or exposure-dependent differences in splenic enlargement. Nevertheless, the study provides a well-defined cohort of patients treated with NAC followed by curative-intent gastrectomy, with standardized CT-based volumetric assessment and radiological response evaluation performed by the same experienced abdominal radiologist. Importantly, RECIST assessment and spleen volumetry were performed in separate reading sessions, and volumetric assessment was conducted independently of clinical, laboratory, and survival outcome data. Furthermore, tissue-based immune parameters were not available, precluding direct assessment of the relationship between splenic changes and the tumor immune microenvironment.

In addition, the cohort was restricted to patients who received NAC and subsequently underwent curative-intent gastrectomy. Therefore, the findings may not be generalizable to all patients initiating NAC, particularly those who experienced disease progression, treatment intolerance, or clinical deterioration precluding surgery.

Furthermore, OS was measured from the date of diagnosis, whereas ΔSV% could only be determined after completion of the pre- and post-NAC imaging assessments. Although diagnosis provided a uniform clinical starting point for survival measurement, this temporal difference should be considered when interpreting the association between ΔSV% and OS.

## 5. Conclusions

In conclusion, SV increased markedly and in nearly all patients during NAC for gastric cancer, demonstrating that splenic enlargement is a highly prevalent treatment-associated imaging finding in this setting. However, in this cohort, the magnitude of SV change was not significantly associated with RECIST-defined tumor response or OS, and pre-NAC SV was likewise not associated with OS. These findings suggest that splenic enlargement during NAC may predominantly reflect treatment-related effects rather than antitumor efficacy in this population; however, they do not exclude a potential prognostic role for spleen volume or its treatment-related change in other populations or treatment settings. Further prospective studies incorporating longitudinal hematological parameters, cumulative chemotherapy exposure, and direct or validated surrogate measures of hepatic sinusoidal injury and systemic immune alterations are warranted to clarify the mechanisms and potential clinical significance of these volumetric changes.

## Figures and Tables

**Table 1:** Clinical, treatment, pathological, laboratory, and spleen volume characteristics according to objective tumor response.

Variable	Overall (*n* = 111)	Nonresponders, SD–PD (*n* = 55)	Responders, CR–PR (*n* = 56)	*p* Value
Age, years	60.37 ± 9.73	57.82 ± 9.39	62.87 ± 9.49	**0.006**
Male sex, *n* (%)	77 (69.4)	37 (67.3)	40 (71.4)	0.788
BMI, kg/m^2^	24.61 ± 3.96	24.05 ± 4.20	25.16 ± 3.67	0.141
**ECOG performance status, *n* (%)**				0.195
ECOG 0	42 (37.8)	17 (30.9)	25 (44.6)	
ECOG 1–2	69 (62.2)	38 (69.1)	31 (55.4)	
**Clinical stage, *n* (%)**				0.323
Stage I–II	32 (28.8)	13 (23.6)	19 (33.9)	
Stage III	79 (71.2)	42 (76.4)	37 (66.1)	
**Clinical T stage, *n* (%)**				0.082
cT1–2	13 (11.7)	3 (5.5)	10 (17.9)	
cT3–4	98 (88.3)	52 (94.5)	46 (82.1)	
**Clinical nodal involvement, *n* (%)**				0.661
cN0	21 (18.9)	9 (16.4)	12 (21.4)	
cN+	90 (81.1)	46 (83.6)	44 (78.6)	
**NAC cycles, *n* (%)**				0.598
1–3	8 (7.2)	5 (9.1)	3 (5.4)	
4	87 (78.4)	41 (74.5)	46 (82.1)	
≥5	16 (14.4)	9 (16.4)	7 (12.5)	
**NAC regimen, *n* (%)**				0.818
FLOT	97 (87.4)	49 (89.1)	48 (85.7)	
FOLFOX	10 (9.0)	4 (7.3)	6 (10.7)	
Other	4 (3.6)	2 (3.6)	2 (3.6)	
**Lauren classification, *n* (%)**				0.748
Intestinal	79 (71.2)	38 (69.1)	41 (73.2)	
Diffuse	28 (25.2)	15 (27.3)	13 (23.2)	
Mixed	3 (2.7)	2 (3.6)	1 (1.8)	
Not classifiable (no residual tumor)	1 (0.9)	0 (0.0)	1 (1.8)	
**ypT category, *n* (%)**				**0.005**
ypT0	3 (2.7)	0 (0.0)	3 (5.4)	
ypT1	5 (4.5)	1 (1.8)	4 (7.1)	
ypT2	21 (18.9)	5 (9.1)	16 (28.6)	
ypT3	61 (55.0)	36 (65.5)	25 (44.6)	
ypT4	21 (18.9)	13 (23.6)	8 (14.3)	
**ypN category, *n* (%)**				0.088
ypN0	58 (52.3)	24 (43.6)	34 (60.7)	
ypN1	13 (11.7)	6 (10.9)	7 (12.5)	
ypN2	29 (26.1)	16 (29.1)	13 (23.2)	
ypN3	11 (9.9)	9 (16.4)	2 (3.6)	
Pre-NAC spleen volume, cm^3^	250.59 ± 98.11	253.11 ± 94.82	248.12 ± 102.03	0.682
Post-NAC spleen volume, cm^3^	335.40 ± 126.81	342.04 ± 129.10	328.88 ± 125.34	0.332
Relative change in spleen volume (ΔSV%), %	34.53 ± 15.72	35.03 ± 15.90	34.05 ± 15.66	0.73
Platelet count, ×10^9^/L	218.00 (70.00–514.00)	216.00 (70.00–514.00)	225.50 (90.00–438.00)	0.315
NLR	2.15 (0.08–24.86)	1.96 (0.08–12.34)	2.31 (0.45–24.86)	0.275
CAR	1.09 (0.12–42.10)	1.18 (0.12–42.10)	0.88 (0.12–25.91)	0.308

Data are presented as mean ± standard deviation, median (minimum–maximum), or *n* (%), as appropriate. Objective responders were defined as patients with complete or partial response (CR/PR), and nonresponders as those with stable or progressive disease (SD/PD) according to RECIST 1.1. Fisher’s exact test was used for comparisons of Lauren classification and ypT and ypN categories. The Mann–Whitney U test was used for comparisons of pre-NAC SV, post-NAC SV, and ΔSV% between responders and nonresponders. BMI, body mass index; ECOG, Eastern Cooperative Oncology Group; NAC, neoadjuvant chemotherapy; FLOT, fluorouracil, leucovorin, oxaliplatin, and docetaxel; FOLFOX, fluorouracil, leucovorin, and oxaliplatin; SV, spleen volume; NLR, neutrophil-to-lymphocyte ratio; CAR, C-reactive protein-to-albumin ratio.

**Table 2:** Univariable and multivariable logistic regression analyses of factors associated with objective tumor response.

Variable	Univariable OR (95% CI)	*p* Value	Adjusted OR (95% CI)	*p* Value
**ΔSV%, per 10-percentage-point increase**	0.961 (0.757–1.220)	0.743	0.932 (0.715–1.207)	0.578
**Pre-NAC spleen volume, cm^3^**	0.999 (0.996–1.003)	0.788	—	—
**Age, per year**	1.059 (1.015–1.104)	**0.008**	1.077 (1.027–1.130)	**0.002**
**BMI, kg/m^2^**	1.076 (0.976–1.186)	0.143	1.054 (0.947–1.173)	0.333
**Male sex**	1.216 (0.542–2.729)	0.635	1.228 (0.497–3.035)	0.656
**ASA III–IV**	0.723 (0.342–1.528)	0.396	—	—
**ECOG 1–2**	0.555 (0.255–1.207)	0.137	0.304 (0.120–0.771)	**0.012**
**Clinical stage III**	0.603 (0.262–1.386)	0.233	0.615 (0.249–1.519)	0.292
**Clinical nodal involvement**	0.717 (0.275–1.870)	0.497	—	—
**NAC cycle group**	0.994 (0.442–2.234)	0.988	—	—
**FLOT regimen**	0.735 (0.237–2.276)	0.593	—	—
**NLR**	1.085 (0.920–1.281)	0.331	—	—
**CAR**	0.980 (0.906–1.060)	0.613	—	—

The dependent variable was objective tumor response (CR/PR vs. SD/PD). ΔSV% was modeled per 10-percentage-point increase. The multivariable model included ΔSV%, age, BMI, sex, ECOG performance status, and clinical stage. Reference categories were female sex, ECOG 0, and clinical stage I–II. —, not included in the multivariable model. OR, odds ratio; CI, confidence interval; BMI, body mass index; ASA, American Society of Anesthesiologists; ECOG, Eastern Cooperative Oncology Group; NAC, neoadjuvant chemotherapy; NLR, neutrophil-to-lymphocyte ratio; CAR, C-reactive protein-to-albumin ratio.

**Table 3:** Univariable and multivariable Cox regression analyses of factors associated with overall survival.

Variable	Univariable HR (95% CI)	*p* Value	Adjusted HR (95% CI)	*p* Value
**ΔSV%, per 10-percentage-point increase**	1.041 (0.886–1.243)	0.603	1.031 (0.859–1.236)	0.745
**Pre-NAC spleen volume, cm^3^**	1.001 (0.998–1.003)	0.586	—	—
**Age, per year**	0.993 (0.966–1.020)	0.59	1.007 (0.979–1.035)	0.624
**BMI, kg/m^2^**	0.919 (0.854–0.988)	**0.023**	0.925 (0.857–0.998)	**0.044**
**Male sex**	0.811 (0.462–1.422)	0.464	0.675 (0.372–1.225)	0.196
**ASA III–IV**	0.849 (0.501–1.437)	0.541	—	—
**ECOG 1–2**	1.121 (0.655–1.917)	0.678	—	—
**Clinical stage III**	1.246 (0.690–2.247)	0.466	1.209 (0.660–2.214)	0.538
**Clinical nodal involvement**	1.048 (0.529–2.076)	0.894	—	—
**NAC cycle group**	0.863 (0.483–1.541)	0.618	—	—
**FLOT regimen**	1.360 (0.615–3.007)	0.448	—	—
**NLR**	1.015 (0.932–1.105)	0.734	—	—
**CAR**	1.037 (0.985–1.091)	0.171	—	—
**Objective response (CR/PR)**	0.585 (0.346–0.990)	**0.046**	0.656 (0.372–1.155)	0.144

ΔSV% was modeled per 10-percentage-point increase. The multivariable model included ΔSV%, age, BMI, sex, clinical stage, and objective tumor response. Reference categories were female sex, clinical stage I–II, and nonresponse (SD/PD). —, not included in the multivariable model. HR, hazard ratio; CI, confidence interval; BMI, body mass index; ASA, American Society of Anesthesiologists; ECOG, Eastern Cooperative Oncology Group; NAC, neoadjuvant chemotherapy; NLR, neutrophil-to-lymphocyte ratio; CAR, C-reactive protein-to-albumin ratio.

## Data Availability

The datasets generated and analyzed during the current study are available from the corresponding author upon reasonable request.
